# Restraint stress of female mice during oocyte development facilitates oocyte postovulatory aging

**DOI:** 10.18632/aging.204400

**Published:** 2022-11-26

**Authors:** Ren-Ren Chen, Jia Wang, Min Zhang, Qiao-Qiao Kong, Guang-Yi Sun, Chun-Hui Jin, Ming-Jiu Luo, Jing-He Tan

**Affiliations:** 1 Shandong Provincial Key Laboratory of Animal Biotechnology and Disease Control and Prevention, College of Animal Science and Veterinary Medicine, Shandong Agricultural University, Tai′an 271018, P.R. China

**Keywords:** mice, oocyte, oxidative stress, postovulatory aging, psychological stress

## Abstract

Studies suggest that psychological stress on women can impair their reproduction and that postovulatory oocyte aging (POA) might increase the risk of early pregnancy loss and affect offspring’s reproductive fitness and longevity. However, whether psychological stress during oocyte development would facilitate POA is unknown but worth exploring to understand the mechanisms by which psychological stress and POA damage oocytes. This study observed effects of female restraint stress during oocyte development (FRSOD) on oocyte resistance to POA. Female mice were restrained for 48 h before superovulation, and they were sacrificed at different intervals after ovulation to recover aging oocytes for analyzing their early and late aged characteristics. The effects of FRSOD on aging oocytes included: (1) increasing their susceptibility to activation stimulus with elevated cytoplasmic calcium; (2) impairing their developmental potential with downregulated expression of development-beneficial genes; (3) facilitating degeneration, cytoplasmic fragmentation and apoptosis; (4) worsening the disorganization of cortical granules and spindle/chromosomes; and (5) impairing redox potential with increased oxidative stress. In conclusion, FRSOD impairs oocyte resistance to POA, so that stressed oocytes become aged significantly quicker than unstressed controls. Thus, couples wishing to achieve pregnancy should take steps to avoid not only fertilization of aged oocytes but also pregestational stressful life events.

## INTRODUCTION

Oocytes of mammals, including those of humans, undergo a progressive degeneration process known as postovulatory oocyte aging (POA) if fertilization or activation does not occur timely after maturation *in vivo* or *in vitro*. Studies show that the fertilization of postovulatory aged oocytes can affect development of the embryo [1–3] and induce severe anomalies in offspring [4, 5]. In human beings, epidemiological investigations indicate that POA might increase loss of the early pregnancy [6], cause daughter reproductive disorder, and reduce offspring longevity [7]. Furthermore, POA is considered as a major cause for the decrease in population size in some endangered species of mammals [8]. Because human beings and some mammals may conduct sexual activity on any day during the estrous cycle due to a lack of mechanisms for synchronization between sexual activity and ovulation, there is the possibility that postovulatory aged oocytes are fertilized by freshly ejaculated spermatozoa. Thus, there is an urgent need for the control over POA. However, the mechanisms and influencing factors for POA are largely unclear.

Although many reports indicated that stress of psychological nature on women could impair their reproduction [9–14], the mechanisms for psychological stress to affect reproduction are not very clear. Animal restraint is an experimental method that has been developed to mimic human psychological stress [15, 16]. Recently, it has been reported that female restraint stress diminished the developmental competence of mouse oocytes [17] with increased chromosome abnormalities [18] and impaired histone modifications [19]. However, whether psychological stress on females during the oocyte development stage would facilitate POA is unknown and is worth exploring to reveal the mechanisms by which psychological stress harms the oocyte and the mechanisms for POA.

Oocytes display morphological, cellular and molecular changes during POA. During the early stages of POA, for example, oocytes show increases in the susceptibility to activation stimulus (STAS) [20, 21] and a decrease in developmental potential [2, 22], whereas cytoplasmic fragmentation [3, 23], disorganized cortical granule distribution [24, 25] and abnormal spindle/chromosome morphology [22, 23, 26] were observed during the late stages of POA. On the molecular level, the aged oocytes also showed increased oxidative stress [23] and decreased expression of the antiapoptotic Bcl2 gene [2, 22, 27] and the antioxidant Sirt1 gene [22, 28].

The aim of this study was to observe the impacts of female restraint stress during oocyte development (FRSOD) on oocyte tolerance to POA. Following the female mice were restraint-stressed for forty-eight hours, they were superovulated with equine chorionic gonadotropin (eCG) and human chorionic gonadotropin (hCG). At different times after the hCG injection, the mice were sacrificed to recover aging oocytes. The oocytes obtained were analyzed for various early and late aged characteristics. The results show that FRSOD significantly impaired oocyte’s tolerance to POA, and as a result, stressed oocytes aged much faster than unstressed controls did.

## RESULTS

### Effects of FRSOD on degeneration, STAS and cytoplasmic calcium level of postovulatory aging oocytes

**Figure 1:**
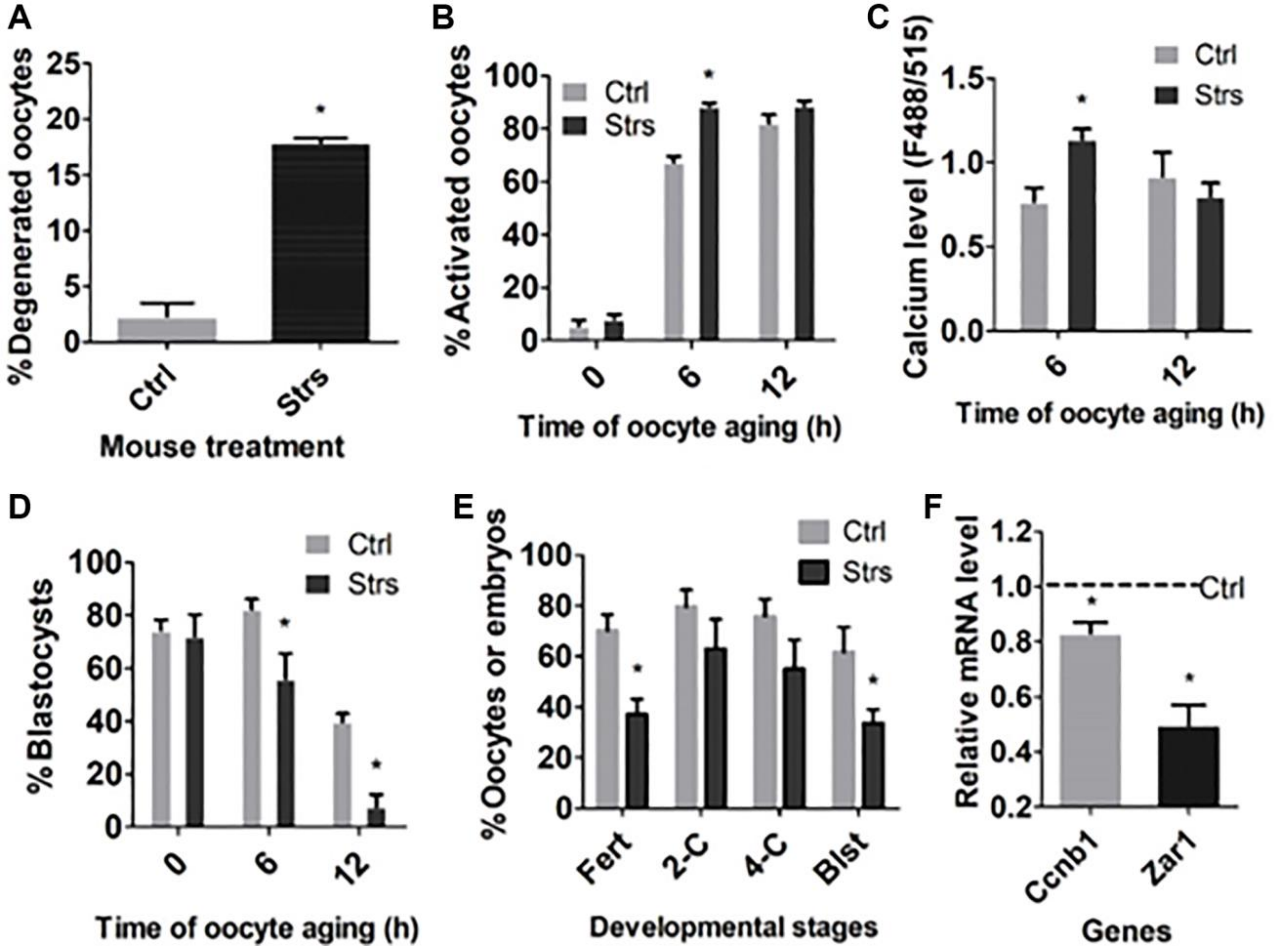
**Effects of FRSOD on degeneration, STAS, cytoplasmic calcium level and developmental potential of postovulatory aging oocytes.** Restraint-stressed (Strs) and unstressed control (Ctrl) mice were killed at 13, 19 and 25 h after hCG injection to recover oocytes aging for 0, 6 and 12 h, respectively. (**A**) Shows percentages of degenerated 12 h-aged oocytes. Each treatment was repeated 3 times with each replicate including 60–70 oocytes. (**B**) Shows percentages of ethanol-activated oocytes after aging for different times. Each treatment was repeated 5 to 7 times with each replicate including 25–30 oocytes. (**C**) Shows cytoplasmic calcium levels (Ex488/Em515) in oocytes aging for different times. Each treatment was repeated 3 times with each replicate containing 30 oocytes from 2 mice. (**D**) Shows percentages of blastocysts following Sr2^+^-activation of oocytes aging for different times. Each treatment was repeated 5 to 8 times with each replicate including 25–30 oocytes. (**E**) Shows rates of fertilization and 2-cell, 4-cell and blastocyst embryos following insemination of 12 h-aged oocytes. Each treatment was repeated 3 times with each replicate including 40–50 oocytes and semen from one male mice. (**F**) Shows gene expression measured by RT-qPCR in 12 h-aged oocytes. Each treatment was repeated 3 times with each replicate including 30–60 oocytes from 2 mice. Values of control oocytes were set to one (dotted line), and the values in stressed oocytes were expressed relative to it. ^*^Indicate significant difference (*P* < 0.05) from values in control mice.

To examine the impacts of FRSOD on degeneration, STAS and ooplasm calcium level of postovulatory aging oocytes, restraint-stressed and unstressed control mice were sacrificed at 13, 19 and 25 h following hCG administration to recover oocytes aging for 0, 6 and 12 h, respectively. The oocytes recovered were either examined for degeneration, treated with ethanol to observe STAS, or measured for cytoplasmic calcium levels. When examined 12 h after ovulation, while the percentage of degenerated oocytes was only about 2% in the control group, it was as high as 18% in the stressed group (Figure 1A). Although rates of activation were not different between control and stressed oocytes aging for 0 or 12 h, they increased significantly in stressed oocytes than in control oocytes aging for 6 h (Figure 1B). Similarly, the cytoplasmic calcium level also elevated significantly in stressed oocytes than in control oocytes aging for 6 h, although it did not differ between control and stressed oocytes aging for 12 h (Figure 1C). Thus, FRSOD significantly facilitated degeneration and increased STAS of aging oocytes.

### Impacts of FRSOD on fertilization and developmental potential of postovulatory aging oocytes

To examine the impacts of FRSOD on fertilization and developmental competence of postovulatory aging oocytes, oocytes were recovered at different intervals following ovulation and were either Sr^2+^-activated or fertilized to observe embryo development, or measured by RT-qPCR for mRNA levels of the developmental competence-related genes. Following Sr^2+^-activation, blastocyst rates in 0 h-aged oocytes were high and not different between the control and stressed groups. However, the blastocyst rates in both 6 h- and 12 h-aged oocytes were lower significantly in stressed than in control groups (Figure 1D). Rates of both fertilization and blastulation were lower significantly in stressed oocytes than in control oocytes when insemination was performed 12 h after ovulation (Figure 1E). Proportions of 2-cell embryos and 4-cell embryos in 12 h-aged oocytes were lower as well in stressed mice than in control mice, although the difference did not reach significant levels statistically. Furthermore, expression of the development-beneficial genes, cyclin B1 (*Ccnb1*) and zygote arrest 1 (*Zar1*), was downregulated significantly in stressed oocytes compared to that in control ones following 12-h aging (Figure 1F). Taken together, the data suggest that FRSOD significantly impaired fertilization and developmental competence of POA oocytes.

### Impacts of FRSOD on ooplasm fragmentation and apoptosis of POA oocytes

**Figure 2:**
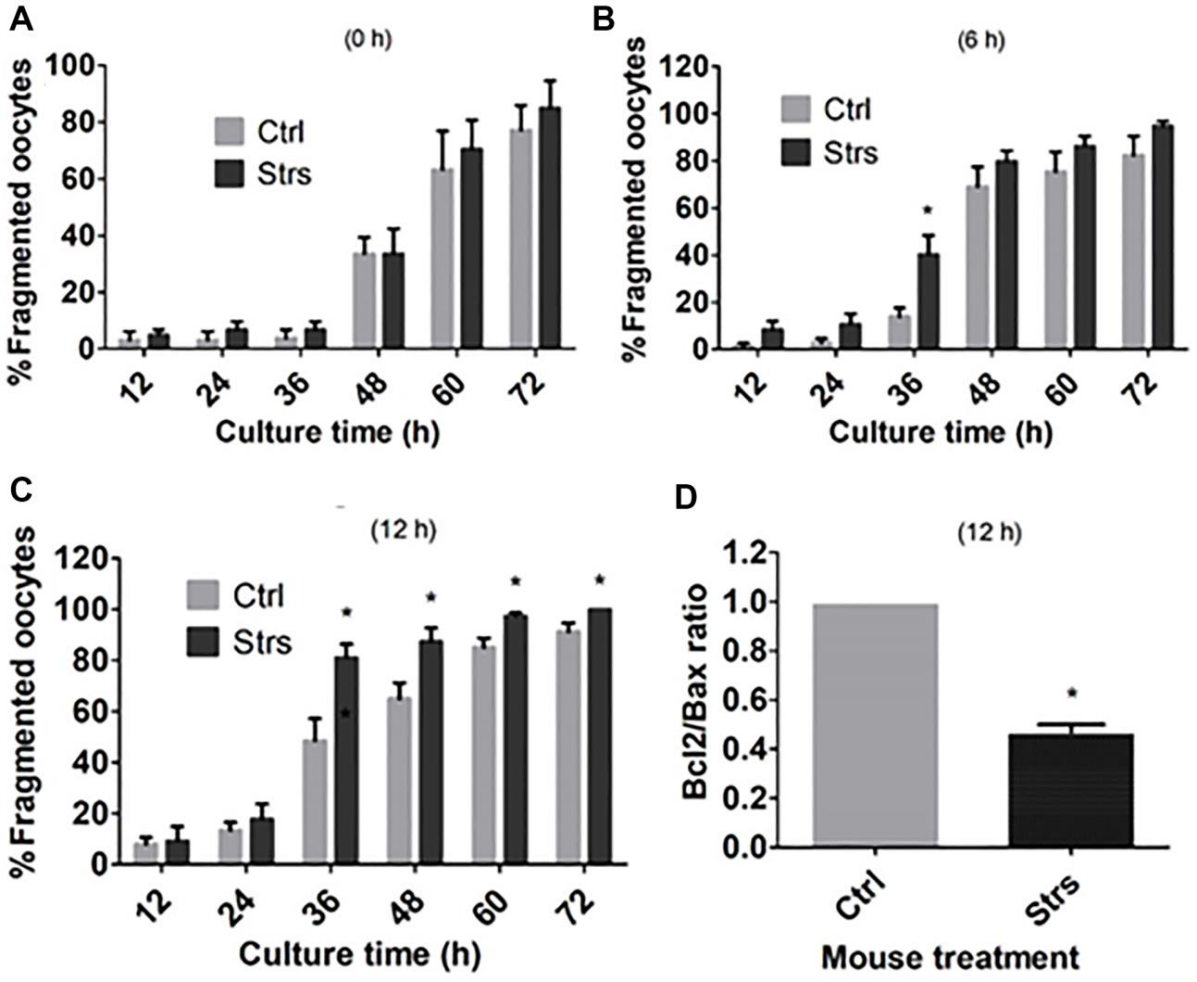
**Effects of FRSOD on cytoplasmic fragmentation and apoptosis of postovulatory aging oocytes.** Restraint-stressed (Strs) and unstressed control (Ctrl) mice were killed at 13, 19 and 25 h after hCG injection to recover oocytes aging for 0, 6 and 12 h, respectively. The oocytes recovered were cultured in CZB medium, and cytoplasmic fragmentation was observed at different times during the culture. Some 12 h-aged oocytes were assayed for expression of Bcl-2 and Bax mRNAs by one-step RT-PCR. (**A**–**C**) Show percentages of fragmented oocytes after oocyte aging for 0, 6 and 12 h, respectively. Each treatment was repeated 6 times with each replicate containing about 20–30 oocytes. (**D**) Compares the ratio of Bcl-2/Bax mRNAs in 12 h-aged oocytes between control and stressed mice. Each treatment was repeated 4 times with each replicate including 30-60 oocytes from 2 mice. ^*^Indicate significant differences (*P* < 0.05) from values in control mice.

To examine the impacts of FRSOD on ooplasmic fragmentation and apoptosis of POA oocytes, oocytes that were recovered at different time intervals following ovulation were incubated in CZB medium, and ooplasmic fragmentation was observed at different hours during the incubation. Fragmentation percentages of the 0 h-aged oocytes did not differ between control and stressed groups when observed at any time points during the culture (Figure 2A). Fragmentation percentages in the 6 h-aged oocytes were higher significantly in stressed groups than in control groups when observed at 36 h of the culture, but the difference between the two groups became insignificant afterwards (Figure 2B). The percentages of fragmentation in 12 h-aged oocytes were higher significantly in stressed mice than in control mice when observed from 36 h to 72 h of the incubation (Figure 2C). Furthermore, the *Bcl2*/*Bax* ratio in 12 h-aged oocytes was lower significantly in stressed mice than in control mice (Figure 2D). Thus, the results suggest that FRSOD facilitated cytoplasmic fragmentation and apoptosis of postovulatory aging oocytes.

### Effects of FRSOD on cortical granules (CGs) distribution and spindle/chromosome morphology of postovulatory aging oocytes

**Figure 3:**
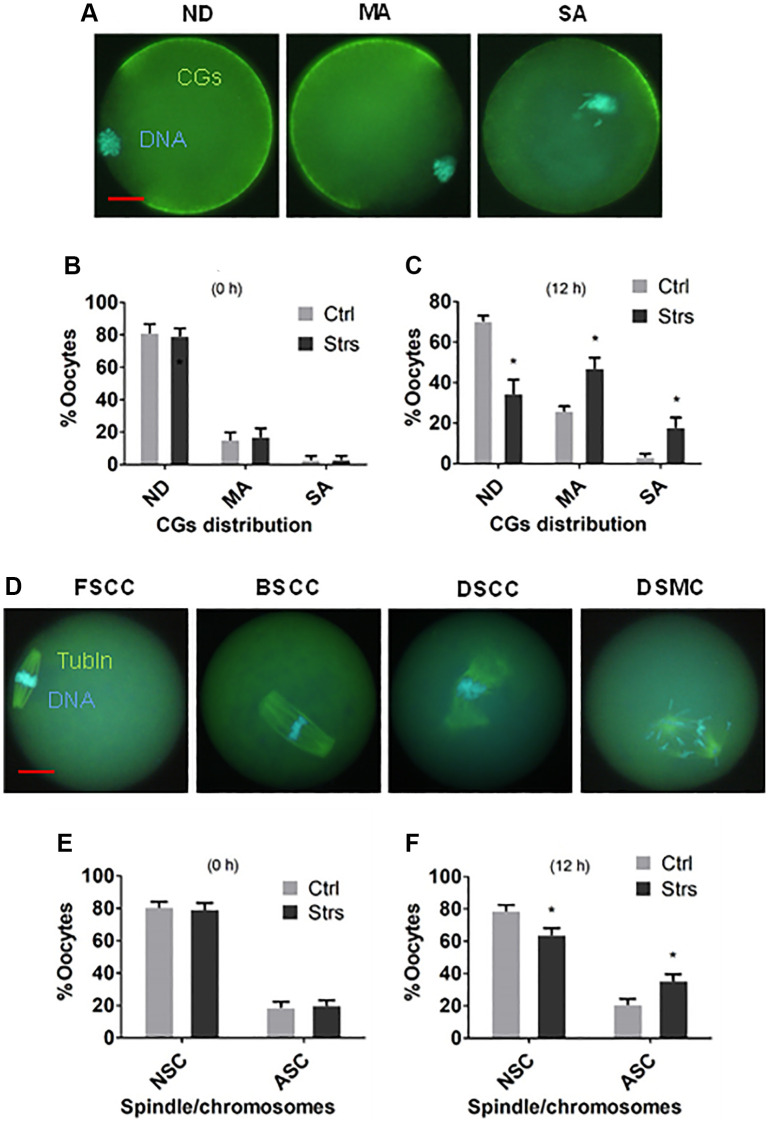
**Effects of FRSOD on cortical granules (CGs) distribution and spindle/chromosome morphology of postovulatory aging oocytes.** Restraint-stressed (Strs) and unstressed control (Ctrl) female mice were killed at 13 and 25 h after hCG injection to recover 0 h- and 12 h-aged oocytes, respectively. The oocytes recovered were observed under a fluorescence microscope after staining with Hoechst 33342 for DNA detection and with antibodies for CGs or a-tubulin (Tubln) detection. The CGs distribution was classified into normal distribution (NB), mildly abnormal (MA) or severely abnormal (SA) distribution. The spindle/chromosome morphology was classified into focused pole spindle (FS), barrel-shaped pole spindle (BS) or disintegrated spindle (DS) with congressed chromosomes (CC) or misaligned chromosomes (MC). While both FSCC and BSCC were considered as normal spindle/chromosome morphology (NSC), DSCC and DSMC were considered as abnormal spindle/chromosome morphology (ASC). (**A**, **B**) Show percentages of oocytes with different CGs distributions after oocytes aged for 0 and 12 h, respectively. Each treatment was repeated 5–6 times with each replicate including 25–35 oocytes from 2 mice. (**C**) Shows micrographs of oocytes with ND, MA and SA types of CGs distribution. (**D**, **E**) Show percentages of oocytes with NSC or ASC spindle/chromosome morphology after oocyte aging for 0 and 12 h, respectively. Each treatment was repeated 5–7 times with each replicate containing 25–30 oocytes from 2 mice. ^*^Indicate significant differences (*P* < 0.05) from values in control mice. The micrographs in (**C** and **F**) Were taken at a magnification of 400×. Bar is 15 μm and is applied to all images.

To observe the effects of FRSOD on CGs distribution and morphology of spindle/chromosome in POA oocytes, oocytes recovered at 0 h and 12 h after ovulation were examined by fluorescence microscopy. The CGs distribution was classified into normal, mildly abnormal and severely abnormal (Figure 3A). In the pattern of normal distribution, CGs were trimly arranged beneath most of the plasma membrane except for the CGs-free area above the spindle/chromosomes complex. In the pattern of mildly abnormal distribution, the plasma membrane area with tidily-aligned CGs decreased significantly due to migration inwards and/or exocytosis. In the severely abnormal pattern, CGs aggregated to form a cap somewhere opposite or near the spindle. In 0 h-aged oocytes, proportions of oocytes exhibiting different CGs distributions were not different between control group and stressed group (Figure 3B). In 12 h-aged oocytes, however, while percentages of oocytes with normal CGs distribution were lower, those with mildly and severely abnormal distribution were higher significantly in stressed group than in control group (Figure 3C).

The morphology of spindle/chromosome was categorized into the focused pole spindle (FS), the barrel-shaped pole spindle (BS) or the disintegrated spindle (DS) with congressed chromosomes (CC) or misaligned chromosomes (MC) (Figure 3D). While FS or BS with CC were considered as normal spindle/chromosome morphology, DS with CC or MC were considered as abnormal spindle/chromosome morphology. In 0 h-aged oocytes, neither percentages of oocytes with normal nor those with abnormal spindle/chromosomes differed significantly between stressed and control groups (Figure 3E). In 12 h-aged oocytes, however, while percentages of oocytes with normal spindle/chromosomes were lower, those with abnormal spindle/chromosomes were higher significantly in stressed mice than in control mice (Figure 3F). The results indicate that FRSOD significantly worsened the disorganization of CGs and spindle/chromosomes during mouse oocyte POA.

### Effects of FRSOD on redox potential of postovulatory aging oocytes

**Figure 4:**
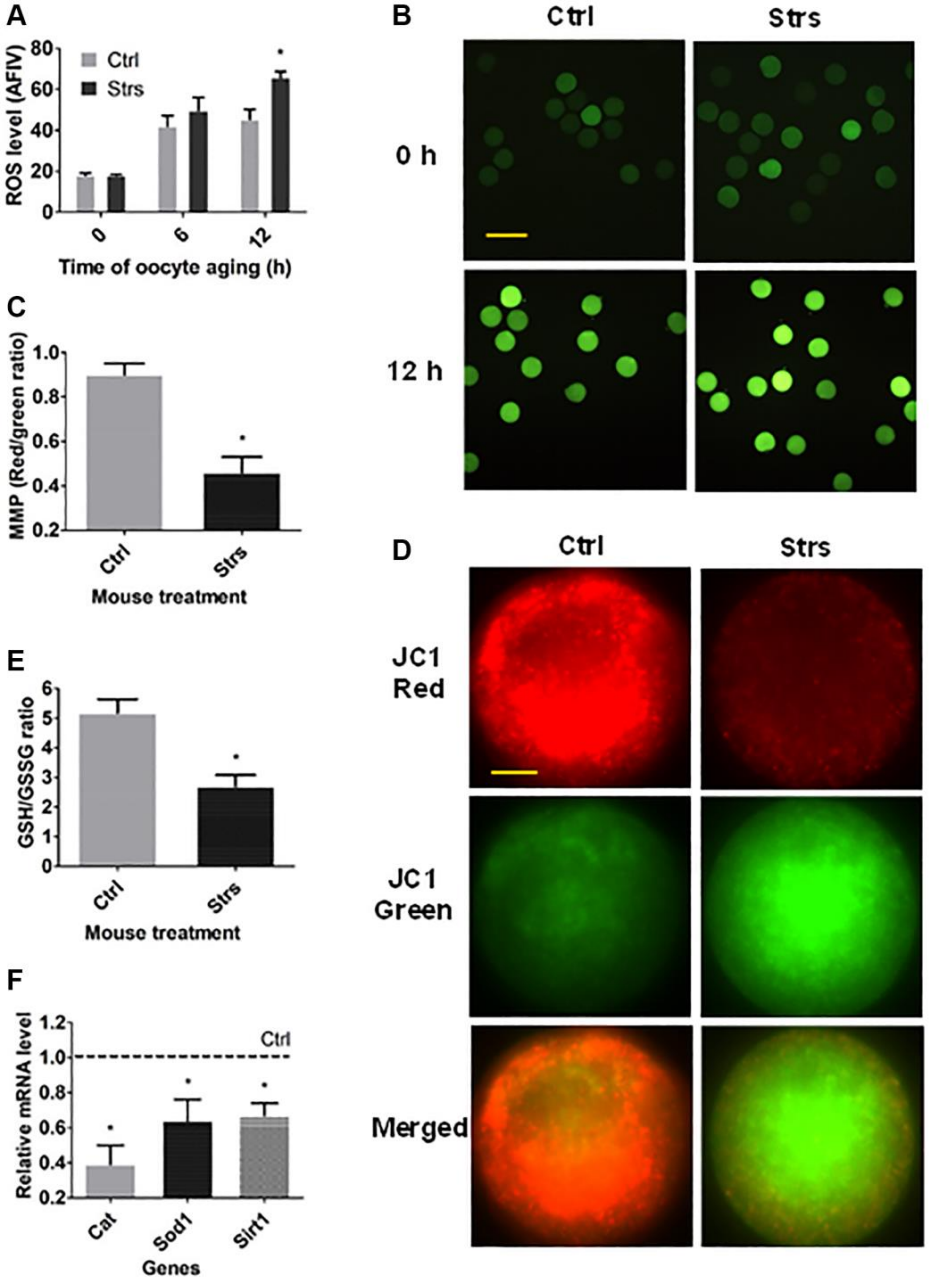
**Effects of FRSOD on ROS level, mitochondrial membrane potential (MMP), reduced glutathione (GSH)/oxidized glutathione (GSSG) ratio and the expression of antioxidant genes in postovulatory aging oocytes.** (**A**) Shows relative ROS level (average fluorescence intensity value, AFIV) in control (Ctrl) and stressed (Strs) oocytes after aging for 0, 6 or 12 h. Each treatment was repeated 3–7 times with each replicate including 30 oocytes from 2 mice. (**B**) Contains fluorescence microscopic images showing the AFIV of ROS in Ctrl or Strs oocytes aging for 0 or 12 h. Bar is 160 μm and is applied to all images. (**C**) Shows MMP (red/green fluorescence intensity) in 12 h-aged oocytes as determined by staining with MMP-specific probe JC-1. (**D**) Contains fluorescence microscopic images showing JC-1 staining intensity in Ctrl or Strs oocytes aging for 12 h. The same oocytes were observed in Cy3 channel (570 nm, red fluorescence) and in FITC channel (512 nm, green fluorescence), respectively. The JC1-red and JC1-green pictures were merged to compared JC1 red and green ratio. Bar is 15 μm and is applied to all images. (**E**) Compares the GSH: GSSH ratio in 12 h-aged oocytes between Ctrl and Strs groups. Each treatment was repeated 3 times with each replicate including about 40 oocytes from 2 mice. (**F**) Shows relative mRNA levels of catalase (Cat), superoxide dismutase 1 (Sod1) and sirtuin 1 (Sirt1) in Ctrl or Strs 12 h-aged oocytes. Each treatment was repeated 3 times with each replicate containing about 30–60 oocytes from 2 mice. ^*^Indicate significant differences (*P* < 0.05) from values in control mice.

To study the impacts of FRSOD on redox potential in POA oocytes, oocytes that had been recovered at 0 h and/or 12 h following ovulation were measured for reactive oxygen species (ROS), mitochondrial membrane potential (MMP), GSH/GSSG ratio, and expression of antioxidant genes. In both 0 h- and 6 h- aged oocytes, the ROS level was not different significantly between the stressed group and the control group (Figure 4A and 4B). In 12 h-aged oocytes, however, the level of ROS in stressed mice was higher significantly than that in control mice. Both the MMP (Figure 4C and 4D) and the GSH/GSSG ratio (Figure 4E) of the 12 h-aged oocytes were lower significantly in stressed mice than in control mice. Furthermore, expression of the three antioxidant enzymes examined in 12 h-aged oocytes, including catalase (*Cat*), superoxide dismutase 1 (*Sod1*) and sirtuin 1 (*Sirt1*), was downregulated significantly in stressed group than that in control group (Figure 4F). The data indicate that FRSOD significantly impaired redox potential leading to increased oxidative stress in postovulatory aged oocytes.

## DISCUSSION

The present results demonstrate that FRSOD facilitated POA. Thus, it increased STAS with elevated cytoplasmic calcium, impaired the developmental potential with downregulated expression of development-beneficial genes, facilitated the degeneration, cytoplasmic fragmentation and apoptosis, worsened the disorganization of CGs and spindle/chromosomes, and increased the oxidative stress with decreased MMP and downregulated expression of antioxidant genes. In somatic cells, many studies have found that the oxidative stress could promote cell apoptosis via both the mitochondria-dependent pathway and the mitochondria-independent pathway [29]. Previous reports proposed that oxidative stress might initiate the other abnormalities in aged oocytes. For instance, the oxidative stress could downregulate the activities of MPF and MAPK, disturb the homoeostasis of calcium, cause the dysfunction of mitochondria as well as directly injure important molecules like lipids, proteins and DNA [23]. Treatment with antioxidants significantly improved the quality of aged oocytes via reducing oxidative stress [30–32]. Furthermore, Zhang and coworkers [28] observed that SIRT1, 2, 3 could suppress oxidative stress, and thus, protect mouse oocytes from POA.

All the aging parameters observed in this study, including STAS, blastocyst rates, fragmentation rates, CGs distribution, spindle/chromosome morphology and ROS level, did not differ between stressed and control oocytes when observed immediately after ovulation. However, the difference in these parameters became significant between stressed and control oocytes when observed at 6 h and/or 12 h after ovulation. These results suggest that FRSOD had initiated a latent proapoptotic program in the developing oocytes in the ovary that was executed later during POA. Liang et al. [33] observed that apoptotic percentages and expression of apoptotic genes in cumulus cells were not different between control mice and the restraint-stressed mice before or after culture with SGH (serum, growth factors and hormones), but increased significantly in stressed mice after culture without SGH. Yuan et al. [34] also observed that injection of mice with cortisol started a program of apoptosis in cumulus cells during the development of ovarian oocytes, and this program was executed later during maturation in culture medium without SGH. Furthermore, the current results also suggest that the tolerance to POA can be used as an important indicator for the quality of oocyte.

This study evidenced that FRSOD downregulated expression of cyclin B1 (*Ccnb1*) and zygote arrest 1 (*Zar1*) genes. The *Ccnb1* gene has been identified as *in vitro* embryo production (IVP) parameter of donor cow quality [35], and increased expression of *Ccnb1* is associated with improved oocyte maturation *in vitro* and post-maturation development [36, 37]. In contrast, a decrease in *Ccnb1* expression has been observed in aged porcine oocytes [38, 39]. Furthermore, chronic restraint stress reduced expression of *Ccnb1*, decreased percentage of GVBD and prolonged time of GVBD in maturing mouse oocytes [40]. The abundance of *Zar1* transcripts is a good predictor for developmental potential of immature oocytes [41]. None of the *Zar1* knockout (−/−) oocytes developed to the 4-cell stage after fertilization [42]. The expression of *Zar1* was upregulated in ovine oocytes produced by more appropriate ovarian stimulation protocols [43]. Furthermore, *Zar1* was mis-expressed in aged human oocytes [44]. Because the current results show that FRSOD decreased blastocyst rates of aged oocytes significantly while having no significant effects on percentages of 4-cell embryos, it is suggested that ZAR1 is required for development not only before but also after zygotic genome activation.

In this study, oocytes with only PB2 without PB1 as well as those with both PB2 and PB1 were considered fertilized after insemination of 12 h-aged oocytes. It has been reported that over 70% PB1 disappeared in mouse oocytes by 12 h after ovulation [45]. Rates of fertilization judged according to this criterion were 70.2% and 37.2%, and percentages of oocytes with normal CGs distribution were 70.6% and 34.7%, respectively, in control and stressed 12 h-aged oocytes. Thus, the percentages of fertilized oocytes were closely correlated with percentages of oocytes with CGs normal distribution, suggesting that (a) our criteria for fertilization judgement were correct and (b) FRSOD impairs fertilization of aging oocytes via facilitating a premature exocytosis of CGs.

In summary, the present results demonstrated that FRSOD significantly facilitated POA. Thus, FRSOD initiated a latent proapoptotic program during the development of ovarian oocytes, and the program was carried out later after ovulation to facilitate POA. During the execution of the FRSOD-initiated apoptotic program in aging oocytes, as an initiator, the increased oxidative stress might promote the early and late anomalies observed in aged oocytes. This suggests that antioxidant measures may be taken to slow down the progress of the stress-facilitated oocyte aging. Because early studies have shown that POA increases the risk of early pregnancy loss [6], causes daughter reproductive disorder, and reduces offspring longevity [7], the current data have important implications for humans. Thus, women who plan to get pregnant not only should take steps to avoid fertilization of aged oocytes, but also, they should take measures to reduce pregestational stressful life events and/or adopt antioxidant measures to slow down the progress of the stress-facilitated POA. Furthermore, the present results suggest that psychological stress, particularly the chronic ones, might cause ovarian aging, as a relationship between stressful life events and premature ovarian failure has been reported in human beings [46, 47]. Thus, this issue should be addressed in future studies using the restraint stress mouse model.

## MATERIALS AND METHODS

This study was conducted according to the relevant guidelines and regulations. The care and handling of mice were carried out fully in accordance with the guidelines reviewed and approved by the Animal Care and Use Committee of the Shandong Agricultural University, P.R. China (Permit number: SDAUA-2001-001). We bought all the chemicals and reagents from the Sigma Chemical Co., if not otherwise specified.

### Management and treatment of mice

We raised the Kunming mice in a mouse room under a photoperiod of 14-h light and 10-h darkness (Lights were off at 20:00 h). We used the female mice 8 to 12 weeks after birth. We placed an mouse in a micro-cage for restraint treatment, and the treatment lasted for 48 h. We made the micro-cage by ourselves, and placed it in an ordinary mouse cage. Whin the micro-cage, the mouse can move back and forth to some degree, but it cannot turn around. Previous studies in this lab have shown that this restraint treatment had no impact on the intake of food and water [17]. Mice for controls were raised in the ordinary cages while the experimental mice were restrained in the micro-cage.

### Superovulation of mice and collection of oocytes

Immediately after the restraint treatment, we superovulated the mice by intraperitoneal injection of 10 IU of eCG (Ningbo Hormone Product Co., Ltd., China) and 10 IU of hCG (Ningbo Hormone Product Co., Ltd.) at 48-h intervals. We killed the restraint-stressed mice and unstressed control mice at 13, 19 and 25 h after hCG injection to obtain oocytes aging for 0, 6 and 12 h, respectively. In most cases (except for the fertilization experiment), the oocytes obtained were denuded of cumulus cells and only those without degeneration signs were used for further analysis. Signs of degeneration included shrinkage, lysis or fragmentation of the ooplasm.

### Activation of oocytes and culture of embryos

To observe STAS, we first treated cumulus-free oocytes at room temperature for 5 min in M2 medium containing 10% ethanol. We then cultured the oocytes in CZB medium at 37.5°C in a humidified atmosphere with 5% CO_2_ in air for 6 h. To test oocyte developmental competence, we activated oocytes by 6 h incubation in a calcium-free CZB medium with 10 mM SrCl_2_ and 5 mg/ml cytochalasin B. After the activation culture, oocytes were examined for activation and those displaying one or two pronuclei, or having two cells each with a nucleus, were judged as activated. We then cultured the Sr^2+^-activated oocytes at 37.5°C in the regular CZB medium (25–30 oocytes /100 μl medium) in humidified air with 5% CO_2_. We examined 4-cell development at 48 h of culture, and at the same time, transferred them to CZB medium containing 5.55 mM glucose and cultured further. We examined blastocyst rates at 96–108 h of the culture. Percentages of blastocysts were calculated from activated oocytes.

### Calcium imaging

We incubated cumulus-free oocytes at 37°C for 20 min in the Hepes-buffered CZB medium supplemented with 0.02% pluronic F-127 and 1 μM Fluo-2 AM, to load the Ca^2+^ probe. We made drops of Hepes-buffered CZB in a Fluoro dish (FD35-100, World Precision Instruments), and then, covered the drops using mineral oil. We then placed oocytes in the drops and examined them at 37°C using an inverted microscope of Leica DMI 6000. For excitation, we used a Fluo-2 fluorescence module with fluorescence excitation and emission wavelengths being 488 and 515 nm, respectively. To calculate the ratio of F488/515, we used a calcium imaging module of Leica LAS-AF. We monitored oocytes for 5 min and recorded the ratio of F488/515 to stand for the ooplasmic calcium concentration.

### *In vitro* fertilization and embryo culture

To collect cauda epididymis and vas deferens, we euthanized male mice (8–12 weeks after birth) by cervical dislocation. By using the edge of an injection needle, we cut the recovered cauda epididymis and vas deferens several times and squeezed them in M2 medium to get sperm masses. We placed the sperm masses in a 1-ml drop of T6 medium supplemented with 10 mg/ml BSA to incubate for 15 min at 37°C. After the incubation, we collected the sperm suspension and measured the sperm concentration. We adjusted the sperm concentration using the same medium to 2 to 4 × 10^7^ sperm/ml, and incubated the spermatozoa for 1.5 h to capacitate them. We then washed oocytes in the fertilization medium (T6 with 20 mg/ml BSA), and put about 30 oocytes in a 150-μl drop of fertilization medium. We placed the capacitated sperm in the fertilization drop to produce about 1 × 10^6^ /ml of sperm concentration. After 6-h fertilization incubation, oocytes were striped of cumulus cells and attaching sperm, and then fixed for 20 min in 4% paraformaldehyde. We stained the fixed oocytes for 5 min in 10 μg/ml Hoechst 33342 before observation for fertilization using a fluorescence microscope. We judged the oocytes with two pronuclei and the second polar body (PB2) and/or first polar body (PB1) as fertilized in this study. At the end of the 6-h fertilization incubation, we cultured the inseminated oocytes in regular CZB medium, and we transferred oocytes to CZB medium containing 5.55 mM glucose at 48 h of the culture. At 96 h of the culture, we examined oocytes for blastocyst rates. Percentages of blastocysts were calculated from inseminated oocytes.

### One-step RT-qPCR

We used the Cell Amp Direct Prep Kit for reverse transcription (RT)-PCR (Real Time) and Protein Analysis (TaKaRa) to treat cumulus-free oocytes to extract RNA. We used One Step TB Green PrimeScript PLUS RT-PCR Kit (Perfect Real Time) for real-time PCR analysis of RNA. Table 1 shows the gene-specific primers used. We normalized gene expression to the internal control of *H2afz*. By using the ^ΔΔ^CT method, we expressed all the values relative to calibrator samples.

**Table 1:** Sequence of primers used in this study.

**Genes**	**Primer sequence (5′—3′)**
*H2afz*	F: ACAGCGCAGCCATCCTGGAGTA
	R: TTCCCGATCAGCGATTTGTGGA
*Bcl-2*	F: TTCGGGATGGAGTAAACTGG
	R: TGGATCCAAGGCTCTAGGTG
*Bax*	F: TGCAGAGGATGATTGCTGAC
	R: TGGATCCAAGGCTCTAGGTG
*Ccnb1*	F: CCGAGAACTGCTCTTGGAGACATTG
	R: TCAGGTTCAGGCTCAGGCTCAG
*Zar1*	F: GACGCCTCGGTGCAGTGTTC
	R: CACAGAAGGTCACGGACGAGAAC
*Cat*	F: GCGGATTCCTGAGAGAGTGG
	R: GAACGGCAATAGGGGTCCTC
*Sod1*	F: CGGTGAACCAGTTGTGTTGT
	R: AGTCACATTGCCCAGGTCTC
*Sirt1*	F: TATCTATGCTCGCCTTGCGG
	R: CGGGATATATTTCCTTTGCAAACTT

### Assessment of oocyte cytoplasmic fragmentation

We recovered oocytes at different time points after hCG administration, removed cumulus cells and cultured the cumulus-free oocytes in CZB medium. At different time intervals during the culture, we examined cytoplasmic fragmentation under a phase-contrast microscope. We considered the oocytes with a moderately granulate cytoplasm and an intact first polar body as unfragmented, but considered those oocytes showing more than two asymmetric cells as fragmented.

### Detection of cortical granules (CGs) and spindle/chromosomes by immunofluorescence microscopy

We first fixed the cumulus-free oocytes for 30 min with 4% paraformaldehyde in M2. Secondly, we removed zona pellucida from oocytes by treatment with 0.5% protease for 10 s (for CGs detection only). Thirdly, we permeabilized oocytes in M2 with 0.1% Triton X-100 at 37.5°C for 5 min. Fourthly, we blocked oocytes at 37.5°C for 1 h in M2 with 3% BSA (for spindle/chromosomes only). To detect CGs, we incubated the blocked oocytes for 30 min in M2 with 100 μg/ml of FITC-labeled lens culinaris agglutinin. To stain tubulin, we incubated the blocked oocytes with FITC-conjugated anti-α-tubulin monoclonal antibodies (1:50) at 37°C for 1 h. To stain chromatin, we incubated the oocytes with 10 μg/ml Hoechst 33342 for 5 min. Negative control samples were processed with the primary antibody omitted. We observed the stained oocytes with a Leica fluorescence microscope.

### Measurement for reactive oxygen species (ROS)

To determine the ROS level in oocytes, we measured H_2_O_2_ levels via staining with DCHFDA (2′,7′-dichloro-dihydro-fluorescein diacetate). We diluted the 1-mM DCHFDA stock solution to 10 μM using M2 medium, and we used the resultant solution to stain cumulus-free oocytes. After a 10-min staining at 37°C, we washed the oocytes in M2, placed them on a slide, and observed them under a Leica fluorescence microscope. We obtained the fluorescence by excitation at 488 nm. To ensure data consistency, we took all the pictures using fixed parameters of microscope. We used the Image-pro Plus software to analyze the fluorescence intensity value of each oocyte.

### MMP (Mitochondrial membrane potential) measurement

We used an MMP detection (JC-1) kit (Beyotime Biotechnology Research Institute, China) to detect MMP. We washed the cumulus-free oocytes thrice in M2 and placed them in a drop of working solution (100 μl M2 and 100 μl JC-1 dye). The oocytes were then cultured for 25 min at 37°C. Then, we washed the oocytes thrice in a JC-1 staining buffer, and observed them using a laser scanning confocal microscope. We observed the same oocyte through both the Cy3 channel (red fluorescence) and the FITC channel (green fluorescence). We detected the aggregate JC-1 (red fluorescence) at 570 nm of emission wavelength, and detected the monomeric JC-1 (green fluorescence) at 512 nm. We calculated the ratio of aggregated/monomeric JC-1 to stand for MMP level. We considered a decrease in red/green JC-1 ratio as an indicator for mitochondrial depolarization.

### Intra-oocyte glutathione assay

We used a commercial assay kit (Beyotime, S0053, Beyotime Institute of Biotechnology) to spectrophotometrically determine concentrations of total glutathione (GSX) and oxidized glutathione (GSSG). We mixed about 40 cumulus-free oocytes with 30 μl of protein scavenger and vortexed them for 5 min. Then, we froze the mixture at −80°C for 3 min and thawed thrice it at room temperature. We conducted centrifugation of the mixture (10,000 g, 0 min, 4°C) and collected the supernatant for measurement or stored it at −80°C before measurement. We used the 5,5′-dithiobis (2-nitrobenzoic acid) (DTNB)-GSSG reductase-recycling assay to measure the concentration of GSX. We also measured 0.5, 1, 2, 5, 10 and 15 μM GSX standards as well as sample blank with no GSX. We obtained absorbance at 405 nm using a spectrophotometer (Beckman Coulter DU 800). We divided the GSX amount by the number of oocytes in each sample to obtain the intra-oocyte concentration of GSX (pmol/oocyte). We calculated the values of reduced glutathione (GSH) using the difference between GSX and GSSG for each oocyte.

### Data analysis

Each treatment contained at least three replicates in all our experiments. Arc sine transformation was first performed for the percentage data. We then analyzed the data using ANOVA when there were more than two groups in each measure, or using independent sample *t*-test when there were only two groups in each measure. We located the differences by carrying out the Duncan multiple comparison test. We used the software of SPSS 11.5 (SPSS Inc.) to perform all the data analyses. We express data as mean ± SEM, and difference was judged as significant when *P* < 0.05.
